# Inferential modeling of 3D chromatin structure

**DOI:** 10.1093/nar/gkv100

**Published:** 2015-02-17

**Authors:** Siyu Wang, Jinbo Xu, Jianyang Zeng

**Affiliations:** 1 Department of Automation, Tsinghua University, Beijing 100084, P.R. China; 2 Toyota Technological Institute at Chicago, 6045 S Kenwood, IL 60637, USA; 3 Institute for Interdisciplinary Information Sciences, Tsinghua University, Beijing 100084, P.R. China; 4 MOE Key Laboratory of Bioinformatics, Tsinghua University, Beijing 100084, P.R. China

## Abstract

For eukaryotic cells, the biological processes involving regulatory DNA elements play an important role in cell cycle. Understanding 3D spatial arrangements of chromosomes and revealing long-range chromatin interactions are critical to decipher these biological processes. In recent years, chromosome conformation capture (3C) related techniques have been developed to measure the interaction frequencies between long-range genome loci, which have provided a great opportunity to decode the 3D organization of the genome. In this paper, we develop a new Bayesian framework to derive the 3D architecture of a chromosome from 3C-based data. By modeling each chromosome as a polymer chain, we define the conformational energy based on our current knowledge on polymer physics and use it as prior information in the Bayesian framework. We also propose an expectation-maximization (EM) based algorithm to estimate the unknown parameters of the Bayesian model and infer an ensemble of chromatin structures based on interaction frequency data. We have validated our Bayesian inference approach through cross-validation and verified the computed chromatin conformations using the geometric constraints derived from fluorescence *in situ* hybridization (FISH) experiments. We have further confirmed the inferred chromatin structures using the known genetic interactions derived from other studies in the literature. Our test results have indicated that our Bayesian framework can compute an accurate ensemble of 3D chromatin conformations that best interpret the distance constraints derived from 3C-based data and also agree with other sources of geometric constraints derived from experimental evidence in the previous studies. The source code of our approach can be found in https://github.com/wangsy11/InfMod3DGen.

## INTRODUCTION

The existence of regulatory DNA components in the genomes of eukaryotic cells has been detected and widely known for decades, but details of the long-range interactions between these exact genomic loci remain elusive. Evidence has shown that long-range interactions between genomic regions, such as promoters and enhancers, may correspond to close spatial proximity (1–3). Thus, understanding the 3D structures of chromosomes can provide important hints toward decoding the mechanisms of gene regulation and chromatin packing, as well as DNA replication, repair and modification (4,5).

In the absence of experimental data, early work on chromatin structure modeling mainly focused on building up a theoretical model to describe the physical property of chromatins based on known knowledge on polymer physics (6). In these models, the chromatin fibers were regarded as a polymer chain and typical features of DNA loops were investigated using molecular dynamics (MD) simulation or Brownian simulation (7–10). Different polymer models for chromatin structures have been proposed, such as random-walk/giant-loop model (11), multiloop-subcompartment model (12,13), random loop model (14) and dynamic loop model (15). These physical models heavily depend on the correctness of the energy function used in the simulation (13). Entropy of conformations was also taken into consideration in some occasions (16–18).

In addition to the theoretical derivations of chromatin structure models, several experimental methods have been developed to study chromosomal architectures. In the early stages, such experiments were conducted mainly through microscopic techniques, typically the 3D fluorescent *in-situ* hybridization (FISH) experiments. By taking advantage of fluorescent DNA probes, the 3D FISH methods can measure the end-to-end physical distances between certain genomic loci. Although providing useful distance restraints for investigating long-range chromatin interactions, the 3D FISH methods are limited by their low throughput.

In recent years, the advent of the chromosome conformation capture (3C) technique and its derivatives has revolutionized the field of studying spatial organizations of chromosomes (19). The 3C-based methods can provide the genome-wide measurements of interaction frequencies between genomic loci close in 3D space (19). These high-throughput 3C-based experimental data provide valuable information to investigate the high-resolution chromosomal conformations. With the rapid development of the 3C-based experimental methods, numerous computational approaches have been proposed to model the 3D chromatin structures from interaction frequency data (20–22). The majority of these approaches (23,24) transformed interaction frequency data derived from 3C-based experiments to local distance constraints, and then formulated the chromatin structure modeling problem into a distance geometry framework, which aimed to compute the 3D coordinates of a set of genomic loci subject to these local distance constraints. The distance geometry framework has been widely used to solve many related engineering problems, such as protein structure determination (25,26) and sensor network localization (27). In most occasions, the distance geometry problems are defined as an optimization task, in which the objective function mainly focuses on minimizing the discrepancy between predicted models and experimental constraints. In some approaches (20,21), additional geometric constraints, such as shape and size of a nucleus, were also included for modeling the 3D organizations of chromosomes. In general, the 3D chromatin conformations are constructed through the minimization of the objective function, which can be performed on many platforms, such as the Integrative Modeling Platform (IMP) or A Mathematical Programming Language (AMPL) (20,28).

To consider uncertainty in experimental data, probabilistic frameworks are often used to formulate the chromatin structure modeling problem (24,29). Among these probabilistic frameworks, the Bayesian approach is probably the most popular one to model chromatin structures from noisy experimental data. In (24), a Bayesian approach that regarded prior probability as an additional constraint was developed, and a Markov chain Monte Carlo (MCMC) method was used to derive the chromatin structures that satisfy the physical distance constraints derived from interaction frequency data. In another Bayesian inference framework (29), a Poisson regression approach was used to derive the contact map constraints of genomic loci from Hi-C data. In addition, two adjacent genomic regions were connected by a rotatable hinge, which allowed one to formulate local structural flexibility into a probabilistic distribution. Currently, the construction of 3D chromatin structures from 3C-based data is still under fast development, and various computational methods from different perspectives have been proposed in the literature (30–35).

In this paper, we aim to integrate other information with 3C-related data to model accurate 3D chromatin structures. In particular, we introduce the inherent conformational energy as prior information and combine it with 3C-related data under a Bayesian inference framework to model 3D chromatin structures. By regarding chromatin fibers as a polymer chain, we naturally define different types of conformational energy terms and systematically unify them with interaction frequency data in a principled way.

As chromosomes exist in a highly dynamical form, especially during interphase, it is not appropriate to describe a chromosome with one single consensus conformation. In this study, we model the 3D spatial arrangement of a chromosome into an ensemble of various candidate structures, each of which is associated with a weight (or probability) to define its likelihood. In addition, we propose an expectation-maximization (EM) based algorithm to estimate the unknown parameters of our Bayesian framework, and infer an accurate ensemble of chromatin structures that best interpret the distance constraints derived from experimental data given our current knowledge on the conformational energy of a chromosome. We have validated the performance of our chromatin structure modeling pipeline via cross-validation and other types of experimental data, such as 3D FISH imaging data. We have further verified the predicted 3D chromatin organizations using the known genetic interactions derived from other studies in the literature. These test results on real 3C-related data have demonstrated that our Bayesian inference approach can provide a practically useful tool to analyze 3C-related data and derive accurate chromatin structures, which will be important for further revealing their genomic features.

## MATERIALS AND METHODS

### Model representation

**Figure 1.**
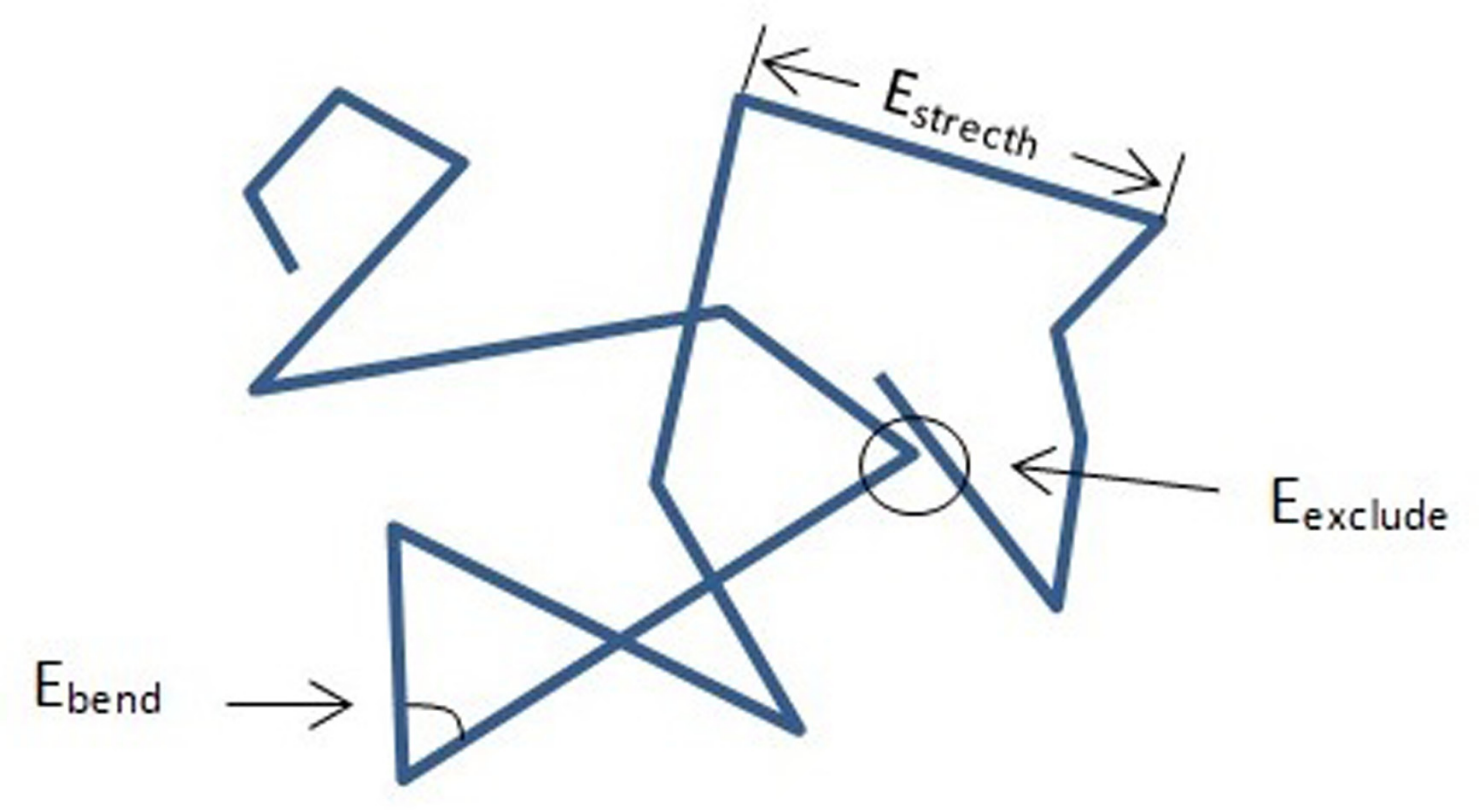
A 2D schematic drawing of the linear segment chain model of a chromatin structure and its conformational energy model.

In our chromatin structure modeling framework, each chromosome is regarded as a linear polymer, i.e. a consecutive line consisting of a number of genomic segments (Figure 1). For example, when modeling the structures of chromosome 1 of yeast, we use a consecutive line of 47 segments, as there are 46 restriction sites cleaved by the HindIII endonucleases on the sequence of this chromosome, and each cutting site acts as an end point of the corresponding segment. Then the aim of our structure modeling pipeline is to derive the 3D coordinates of these end points based on the input interaction frequency data, and thus obtain a complete spatial arrangement of the whole chromosome. Although the way of splitting the whole chromosome into segments is generally considered a coarse-grained representation, in our framework, as each segment corresponds to a genomic unit between two enzyme cutting sites adjacent in sequence, such a representation is probably the most accurate model given current resolution of 3C-related data.

### Defining the conformational energy of chromatin structures

In the literature, a number of theoretical models have been proposed to define the ‘conformational energy’ of a chromatin structure, which describes the physical or statistical potential of a chromatin conformation (13–14,36). Here, we apply several common rules and formulas of a typical polymer model to derive the conformational energy of a given chromatin structure. We first introduce some notation for defining the conformational energy of a chromosome. Given a chromatin structure **S** with a linear chain of *n* segments, we use *l*_*i*_ to represent the length of the *i*-th segment, where 1 ≤ *i* ≤ *n*. We use $\textbf {s}_{j}=\left( x_{j},y_{j},z_{j}\right)$ to represent the spatial position of the *j*-th segment end point, where 1 ≤ *j* ≤ *n* + 1. Meanwhile, the length of each segment is calculated based on the Euclidean distance between two adjacent end points, that is,

(1) \begin{eqnarray*} l_{i}&=& \left| \textbf {s}_{i+1}- \textbf {s}_{i} \right| \nonumber \\ &=&\sqrt{\left( x_{i+1}-x_{i} \right)^{2}+\left( y_{i+1}-y_{i} \right)^{2}+\left( z_{i+1}-z_{i} \right)^{2}}. \end{eqnarray*}

As illustrated in Figure 1 of the paper, the conformation energy of a chromosome **S**, denoted by *E*_*s*_, generally consists of the following three terms:

(2) \begin{equation*} E_{s}=E_{{\rm stretch}}+E_{{\rm bend}}+E_{{\rm exclude}}, \end{equation*}

where *E*_stretch_, *E*_bend_ and *E*_exclude_ represent the ‘stretching’, ‘bending’ and ‘excluding’ energies of chromatin segments, respectively. The details of these three energy terms will be explained below.

First, the stretching energy of chromatin segments, *E*_stretch_, which describes the potential energy corresponding to the stretching force of chromatin fibers, is defined by

(3) \begin{equation*} E_{{\rm stretch}}=\sum _{i=1}^{n}\dfrac{1}{2}k_{s}(l_{i}-l_{i0})^{2}, \end{equation*}

where *k*_*s*_ stands for the ‘bond spring constant’, indicating the stiffness of a chromatin fiber, *l*_*i*_ stands for the length of the *i*-th segment and *l*_*i*0_ stands for the length of a chromatin segment in a state of equilibrium. The bond spring constant is generally proportional to *K*_*B*_*T*, where *K*_*B*_ and *T* represent the ‘Boltzmann constant’ and the absolute temperature, respectively. The packing density of a chromatin fiber is normally supposed to be 130 bp/nm (37). Thus, the parameter *l*_*i*0_ can be defined by $l_{i0}=\frac{l_{is}}{130}$, where *l*_*is*_ represents the total number of base pairs in the chromatin segment.

Second, the bending energy, *E*_bend_, which describes the twisting potential of a chromatin structure, is mainly defined by the the angle between adjacent segments (38):

(4) \begin{equation*} E_{{\rm bend}}=\sum _{i=2}^{n}\frac{1}{2}k_{\theta }\left\langle \textbf {s}_{i+1}-\textbf {s}_{i},\textbf {s}_{i}-\textbf {s}_{i-1} \right\rangle ^{2}, \end{equation*}

where *k*_θ_ stands for the ‘bending constant’, indicating the bending elasticity of a polymer chain, and 〈·〉 measures the torsion angle between these two adjacent segments.

Third, the excluding energy, *E*_exclude_, describing the situation in which two parts of a chromosome cannot be packed too closely due to the repulsion force, is defined based on the Lennard–Jones potential (39). In particular, we use the following formula (34) to define the excluding energy term:

(5) \begin{eqnarray*}
&&E_{{\rm exclude}}= \nonumber\\
&&\left\lbrace \begin{array}{@{}cl@{}} \sum \nolimits _{2\le i+1\le j\le n}^{ }4\varepsilon \left[ \left( \frac{\delta }{d_{i,j}} \right)^{12}-\left( \frac{\delta }{d_{i,j}} \right)^{6}+\frac{1}{4} \right], &d_{i,j}<2^{\frac{1}{6}}\delta {;} \\
0, &{\rm otherwise}, \end{array}\right.
\end{eqnarray*}

where $d_{i,j}=\left| \textbf {s}_{i}-\textbf {s}_{j} \right|$ is the Euclidean distance between two segment end points *s*_*i*_ and *s*_*j*_, and ϵ and σ are parameters.

For most of the parameters in this model, we use the same setting as in (34). The details of these parameter choices are provided in Table 1.

**Table 1.** The parameter setting of our conformational energy model

Parameter	Symbol	Reduced unit	SI unit
Thermal energy	*K_B_T*	1.0	4.1 × 10^−21^*J*
L–J size parameter	δ	1.0	30 nm
L–J energy parameter	ϵ	1.0*K*_*B*_*T*	4.1 × 10^−21^*J*
Bond spring constant	*k*_*s*_	$500\frac{K_{B}T}{\sigma ^{2}}$	$2.3\times 10^{-3}\frac{J}{m^{2}}$
Bending energy constant	*k*_θ_	$4\frac{K_{B}T}{{\rm rad} ^{2}}$	$1.7\times 10^{-20}\frac{J}{{\rm rad}^{2}}$

We choose the same values as in (34) for most of the parameters in the model. L–J stands for Lennard–Jones.

### Bayesian inference of a chromatin structure

In recent years, with the development of the 3C-based techniques for measuring the end-to-end interaction frequencies between genomic loci, numerous computational methods have been proposed to model the 3D structures of chromosomes (20,24,29). Most of these methods formulated the structure modeling problem into an optimization framework, in which the objective was to compute the chromatin conformations that agree with the experimental data (20,23,40). Only a small number of existing approaches (24,29,35) have exploited prior knowledge of chromosomes, such as geometric constraints of a nucleus, the physical or conformational energy of a chromosome, during the structure modeling process. Even when the conformational energy was used to help determine the chromatin structures, the weighting parameter between the conformational energy and the data terms was mainly decided in an ad hoc manner rather than in a principled framework (24). In addition, most of the previous chromatin structure modeling approaches were deterministic (41,42). Due to uncertainty in experimental data, it is more natural and reasonable to formulate the structure modeling problem into a probabilistic framework. Furthermore, due to current limitations in the 3C-based experiments, the interaction frequencies between genomic loci are generally averaged measurements over a population of heterogeneous cells. Thus, it should be more accurate to compute an ensemble of chromatin conformations rather than a single consensus structure from the 3C-based experimental data.

To address the above issues in chromatin structure modeling, here we propose a Bayesian inference approach to systematically integrate the conformational energy with experimental data, and compute an ensemble of chromatin structures that best interpret the distance constraints converted from interaction frequency data. Our approach employs a probabilistic framework to model uncertainty in experimental data, and chooses the parameters in a rational framework. Note that the Bayesian inference approach has been successfully used in determining protein structures from experimental data (43,44). In our problem, the basic formula for chromatin structure modeling according to Bayes’ theorem can be expressed as follows:

(6) \begin{equation*} \Pr ({\bf S}|D)= \frac{\Pr ({\bf S})\Pr (D|{\bf S})}{\Pr (D)}, \end{equation*}

where **S** represents a chromatin structure and *D* stands for experimental observation derived from 3C-related data. In particular, we use the spatial distances converted from 3C-based data. More details on converting interaction frequencies to spatial distances can be found in Supplementary Material S1. In general, $\Pr (D)$ can be considered a constant. The terms $\Pr ({\bf S}|D)$, $\Pr (D|{\bf S})$ and $\Pr ({\bf S})$ are also called ‘posterior probability’, ‘likelihood function’ and ‘prior probability’, respectively. In this section, we mainly focus on the computation of the maximum a posteriori (MAP), which finds a conformation that maximizes the Bayesian formula in Equation (6). In the next sections, we will extend this framework to compute an ensemble of chromatin structures that best interpret the converted distance constraints. In our framework, the likelihood function is defined as

(7) \begin{equation*} \Pr (D|{\bf S})=\prod _{i=1}^{m}\Pr (D_{i}|{\bf S}), \end{equation*}

where *D*_*i*_ represents the *i*-th data record in the interaction frequency data set and *m* stands for the total number of data records from experimental measurements. We follow a commonly accepted assumption in the literature: the spatial distance between two genomic loci is inversely proportional to their interaction frequency (see Supplementary Material S1). Here, we assume that different spatial distance restraints derived from experimental observation between genomic loci are independent to each other. In addition, we apply Gaussian distribution to model experimental noise, that is,

(8) \begin{equation*} \Pr (D_{i}|{\bf S})\sim \frac{1}{\sqrt{2\pi }\sigma }\exp (-\frac{1}{2\sigma ^{2}}(D_{i}^{s}-D_{i})^{2}), \end{equation*}

where $D_{i}^{s}$ represents the back-computed distance in conformation **S** and σ represents the standard deviation of Gaussian noise. Here, we assume that experimental data follows Gaussian distribution. In principle, other distributions, such as Poisson distribution, may also be used to describe the interaction frequency data.

The prior probability $\Pr ({\bf S})$ describes the possibility of conformation **S** based on our prior knowledge. Here, we mainly use the Boltzmann distribution based on the conformational energy derived in the previous section to define the prior probability, that is,

(9) \begin{equation*} \Pr ({\bf S})\sim \exp \left(-\frac{E_{s}}{K_{B}T}\right), \end{equation*}

where *K*_*B*_ denotes the Boltzmann constant, *T* denotes the absolute temperature and *E*_*s*_ denotes the conformational energy of conformation **S** computed using the model described in the previous section.

After substituting Equations (7), (8) and (9) into Equation (6), we have

(10) \begin{eqnarray*} \Pr ({\bf S}|D)\propto \frac{1}{\sigma ^{m}}\exp \left( -\frac{E_{s}}{K_{B}T} \right)\exp \left( -\frac{1}{2\sigma ^{2}}\sum _{i=1}^{m} \left|D_{i}^{s}-D_{i}\right|^{2}\right).\nonumber\\ \end{eqnarray*}

Usually we calculate the logarithm of Equation (10) rather than computing the probabilistic function directly. After taking the logarithm on both sides of Equation (10), we have

(11) \begin{equation*} L({\bf S}|D)=-\frac{E_{s}}{K_{B}T} -\frac{1}{2\sigma ^{2}}\sum _{i=1}^{m} \left(D_{i}^{s}-D_{i}\right)^{2}-m\log \sigma {.} \end{equation*}

In Equation (11), we only take the chromatin conformation **S** as an unknown variable. We can also consider experimental noise (denoted by σ) and the parameter for converting interaction frequencies into spatial distances (denoted by α, see Supplementary Material S1) as unknown variables and apply the Jeffreys prior to describe their prior probabilities, using the same strategy as in (43). Then the logarithm likelihood of Equation (11) can be rewritten as

(12) \begin{equation*} \begin{split} L({\bf S}|D,\sigma ,\alpha )=-\log {\sigma }-\log {\alpha }-\frac{E_{s}}{K_{B}T} \\ -\frac{1}{2\sigma ^{2}}\sum _{i=1}^{m} \left(D_{i}^{s}-D_{i}\right)^{2}-m\log \sigma {.} \end{split} \end{equation*}

The MAP estimation is equivalent to finding the conformation $\textbf {S}^{*}$, and parameters σ* and α* that maximize *L*(**S**|*D*, σ, α). In Equation (12), the factor $\frac{1}{2\sigma ^{2}}$ can also be considered the weighting factor between two terms that represent the conformational energy and the data restraints, respectively. When the conformational energy and the data term are integrated together, it is necessary to ensure that the choice of the weighting factor is appropriate so that both terms can be tuned into the proper order of magnitude. On the other hand, the choice of the weighting factor $\frac{1}{2\sigma ^{2}}$ can also be explained by the magnitude of experimental noise in observation data. When experimental data contain more noise (i.e. with larger σ), we should put a smaller weight $\frac{1}{2\sigma ^{2}}$ for the data term in Equation (12), and vice versa.

### Ensemble modeling of chromatin structures

Most of the previous chromatin structure modeling approaches computed a single unique conformation that best fits experimental data (20,23,41–42). Unfortunately, computing a single conformation with the maximum likelihood (ML) estimation is only an ideal situation without considering many practical factors. In general, the interaction frequencies from 3C-based experiments are the population-averaged measurements over multiple cells in a sample (19). Also, a chromosome generally exists in a highly dynamical form in a nucleus and its shape usually changes during different phases of cell cycle (45). These factors indicate that it is relatively inaccurate to model a chromosome as a single unique structure. In addition, uncertainty in 3C-based experimental data usually makes it more difficult to obtain a single accurate chromatin structure from current 3C-based techniques.

To address the above problems, we compute an ensemble of chromatin structures rather than a single consensus conformation under our Bayesian inference framework. The idea of ensemble structure modeling has been widely used in protein structure modeling, e.g. structure modeling of intrinsically disordered proteins (46) and protein structure determination from nuclear magnetic resonance data (43). A structure ensemble can be regarded as a set of different conformations, each of which is associated with a probability (or weight) that describes its existence possibility. More specifically, a structure ensemble $\mathcal {S}$ of a chromosome can be defined as

(13) \begin{equation*} \mathcal {S}=\left\lbrace \left({\bf S}_{1},w _{1} \right),\left({\bf S}_{2},w_{2} \right),\cdots ,\left({\bf S}_{k},w_{k} \right) \right\rbrace {.} \end{equation*}

In additional, all weights *w*_*i*_ should satisfy

(14) \begin{equation*} \sum _{i=1}^{k} w _{i}=1, \text{and } 0\le w _{i}\le 1, \mbox{ for all } 1\le i\le k, \end{equation*}

where **S**_*i*_ represents individual conformations, *w*_*i*_ represents the corresponding probability (or weight) and *k* represents the total number of conformations in the ensemble. Currently we choose the value of *k* empirically. Basically, the value of *k* should be chosen appropriately so that the diversity of conformational space can be fully explored within the available computational resources. In our tests on the yeast chromosome, the value of *k* is set to be 200.

### An EM based approach for chromatin structure modeling

In our Bayesian inference framework, although our primary goal is to compute individual chromatin conformations and their corresponding weights in the ensemble, there are also several other unknown parameters that need to be estimated, such as experimental noise σ and the exponential factor α for converting interaction frequencies to spatial distances. These additional unknown variables make it more difficult to address the structure modeling problem. In our paper, we apply an EM like algorithm to solve this Bayesian inference problem. EM is an inference algorithm that has been widely used in machine and statistical learning to find the ML or MAP estimate of latent variables (47). In particular, the EM algorithm iteratively and alternately performs two steps, namely the Expectation (E) step and the Maximization (M) step. The E step calculates the expectation of a probabilistic function using the current estimates of unknown parameters and the M step performs the ML or MAP estimates of these parameters using the expected likelihood derived in the proceeding E step (47).

**Figure:**
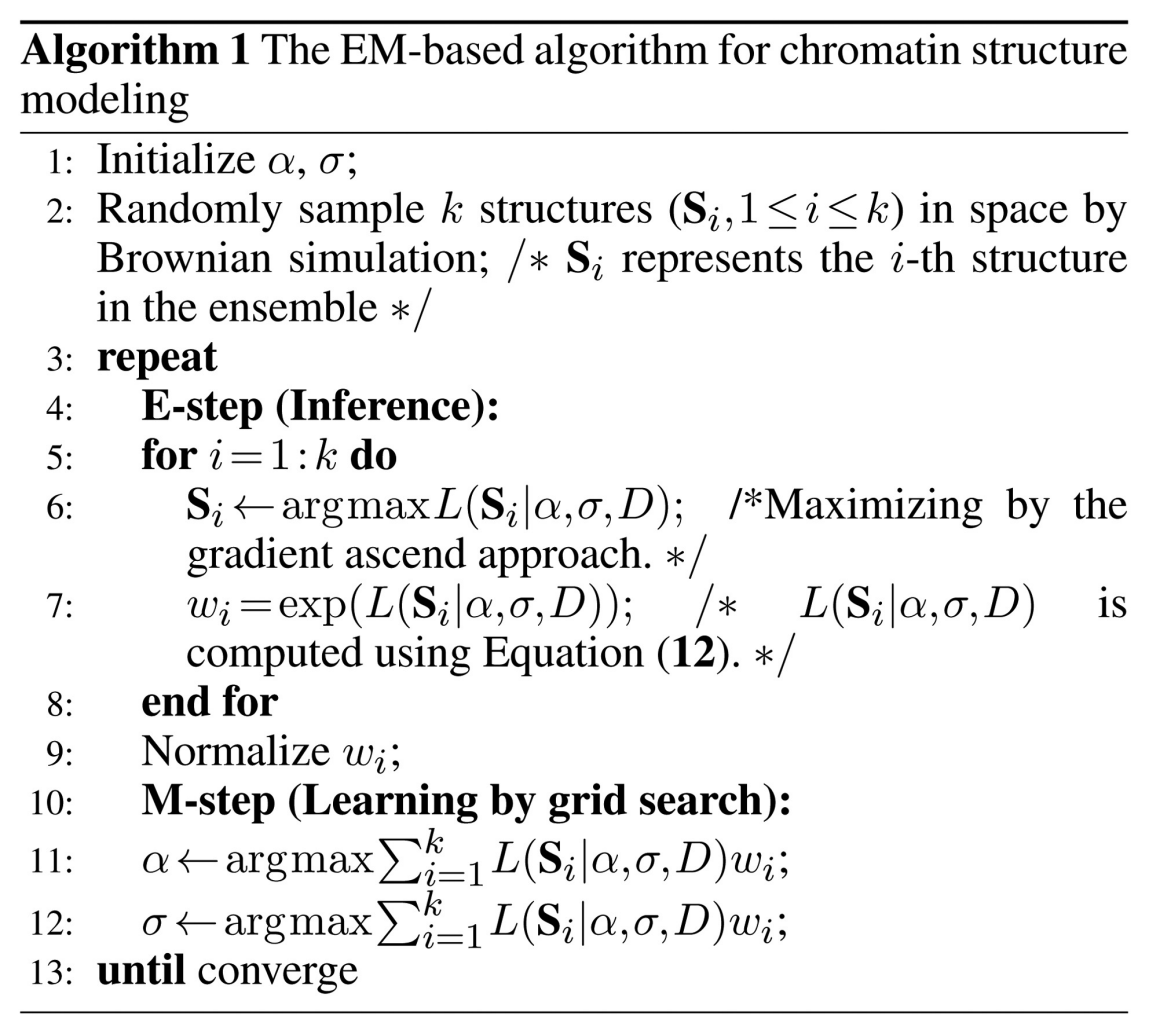


The details of our EM-based method for inferring chromatin structures are provided in Algorithm 1. Initially the algorithm assigns random values to parameters σ and α, which represent experimental noise and the exponential factor for converting interaction frequencies to local distance restraints, respectively. Then the algorithm randomly generates an initial pool of *k* conformations using Brownian simulation. The size of the chromosome (see Supplementary Material S1) is also used as a geometric constraint during the simulation. Note that the Brownian simulation approach has been widely used to produce an ensemble of molecular structures in the literature (46,48–49). In the E step, we first improve the qualities of individual structures in the ensemble by optimizing every structure with the estimated parameters derived in the proceeding steps. The optimization is realized using a gradient ascend approach, in which the corresponding likelihood of each structure (i.e. Equation (12)) is maximized. We then compute the expectation of the likelihood function, which is the conditioned distribution of the structure ensemble {(**S**_*i*_, *w*_*i*_)} given the current estimates of unknown parameters α and σ. For each structure, the corresponding likelihood function is defined in Equation (12). The corresponding weights (or probabilities) of individual conformations are then calculated based on the derived likelihood functions after normalization (Line 9 in Algorithm 1). In the M step, we use a grid search method to find the best estimates of parameters α and σ that alternately maximize the following formula:

(15) \begin{equation*} L= \sum _{i}L({\bf S}_{i}|\alpha ,\sigma ,D)w_{i}. \end{equation*}

We call this function the ‘ensemble likelihood’. In the grid search for estimating the values of parameters σ and α, we divide the possible ranges of parameters into grid points and choose a pair of grid points for parameters α and σ that maximize Equation (15). The ranges and step lengths of the grid search are determined empirically. In particular, a method similar to coarse-grained sampling is used to estimate the possible ranges of the parameters α and σ, that is, we first probe the possible intervals of the parameters using large step lengths, and then use small step lengths to narrow down the ranges. As the relationships of the objective function versus individual parameters show a clear one-peak trend (see Figure 3), the aforementioned process can be done easily. Using the above procedure, we can compute an ensemble of chromatin structures that best interpret the geometric constraints derived from experimental data based on our prior knowledge on the conformational energy of a chromosome.

## RESULTS AND DISCUSSION

### Data acquisition

The chromosome conformation capture (3C) based methods have become a powerful tool for investigating the 3D genome architecture (50–52). In general, the raw data (in the form of sequencing data) can be converted to a heat map of interaction frequencies, which is comprehensible and usually taken as input for chromatin structure modeling (53–55).

Using the genome-wide 3C-based data, we are able to model the 3D architecture of the whole chromosome. Currently, a large amount of raw 3C-based data can be accessible to the public and downloaded from the Gene Expression Omnibus (GEO) repository on the National Center for Biotechnology Information (NCBI) website (http://www.ncbi.nlm.nih.gov/geo/). In particular, we use the data of yeast (41) to study the spatial organizations of its chromosomes. Note that the yeast genome is relatively small (12.1 Mb in total), and thus can be relatively easily and effectively used to validate a chromatin structure construction method on the chromosome scale. In the next sections, we will mainly use the 3C-based data of different chromosomes of yeast as an example to demonstrate our chromatin structure modeling pipeline. There are also other necessary procedures required to preprocess the raw data, such as the correction based on coverage or other information (53) and the mapping from the raw sequence to the genome (55). As these steps are not the main focus of our work, here we directly use the preprocessed interaction frequency heat map as input data to our algorithm.

### The convergence of the EM-based algorithm

**Figure 2.**
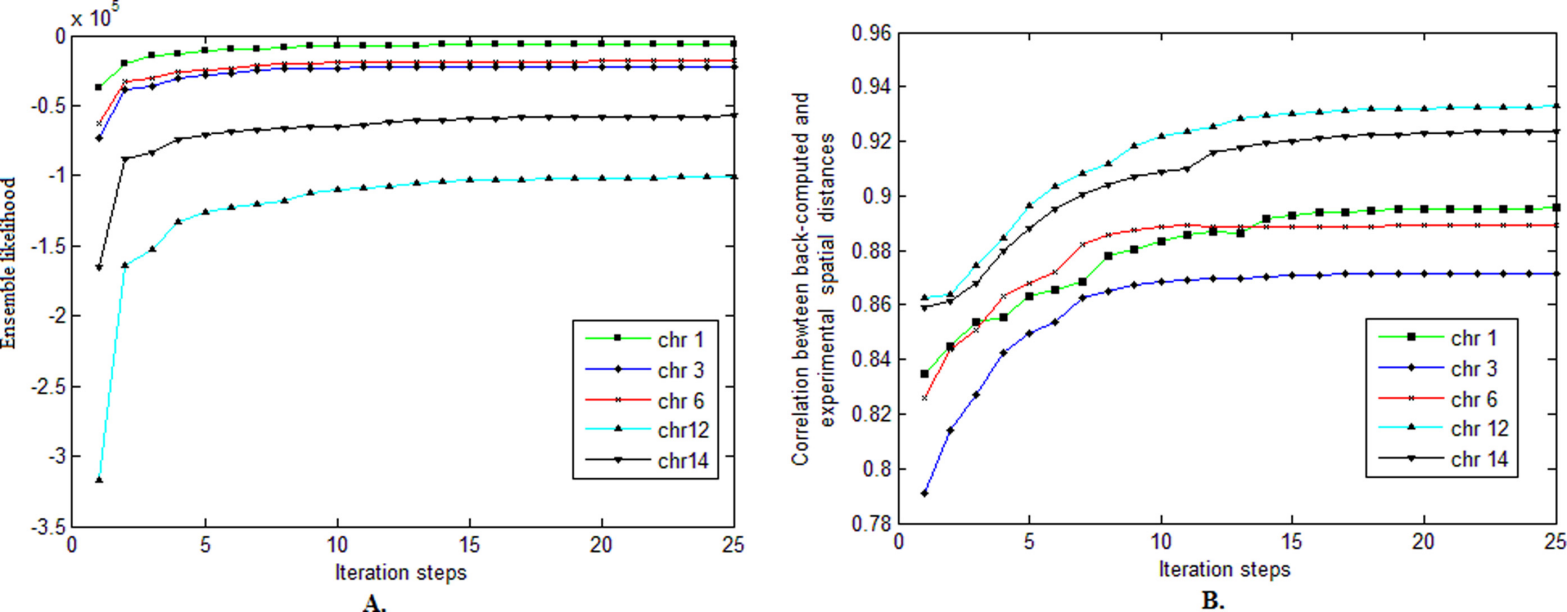
The convergence of our EM-based algorithm. (**A**) The convergence results on the ensemble likelihood. (**B**) The convergence results on the correlation between back-computed and experimental spatial distances. The ensemble likelihood (as defined in Equation (15)) of individual chromosomes may converge to different values because different chromosomes usually have different parameters (e.g. genomic lengths). When computing the correlation between back-computed and experimental spatial distances, the ensemble-averaged values were used.

We first checked the convergence of our EM-based algorithm for solving the structure inference problem. We tested the algorithm on the 3C-based data of different chromosomes of yeast. We examined the ensemble likelihood (as defined in Equation (15)) and the correlation between back-computed and experimental restraints (i.e. local distance restraints derived from predicted models and interaction frequencies in experimental data, respectively). As shown in Figure 2, the ensemble likelihood converged to a relatively stable value within a small number of iteration steps. After 10–15 iteration steps, the correlation between spatial distance restraints back-computed from the predicted models and converted from experimental data also converged to a stable state. It may appear that our algorithm converge quickly. For example, after 10–15 iteration steps, the correlation can converge to a stable state. But in fact, each iteration step may require a large number of optimization sub-steps (see the pseudocode of Algorithm 1). In particular, it may take the gradient ascend approach (Line 6 in Algorithm 1) thousands of optimization sub-steps to compute the maximum value in the E-step. Also, the grid search (Lines 11–12 in Algorithm 1) may run for a while to find the optimum in the M-step, depending on the ranges and step lengths used in the search. In our tests on the yeast chromosomes, it took our algorithm 30–50 h to converge. Note that tests on the 3C-based data of different chromosomes usually achieved different convergence states. This was expected, as different chromosomes typically have different parameter settings of parameters (e.g. various genomic lengths).

### Parameter selection

**Figure 3.**
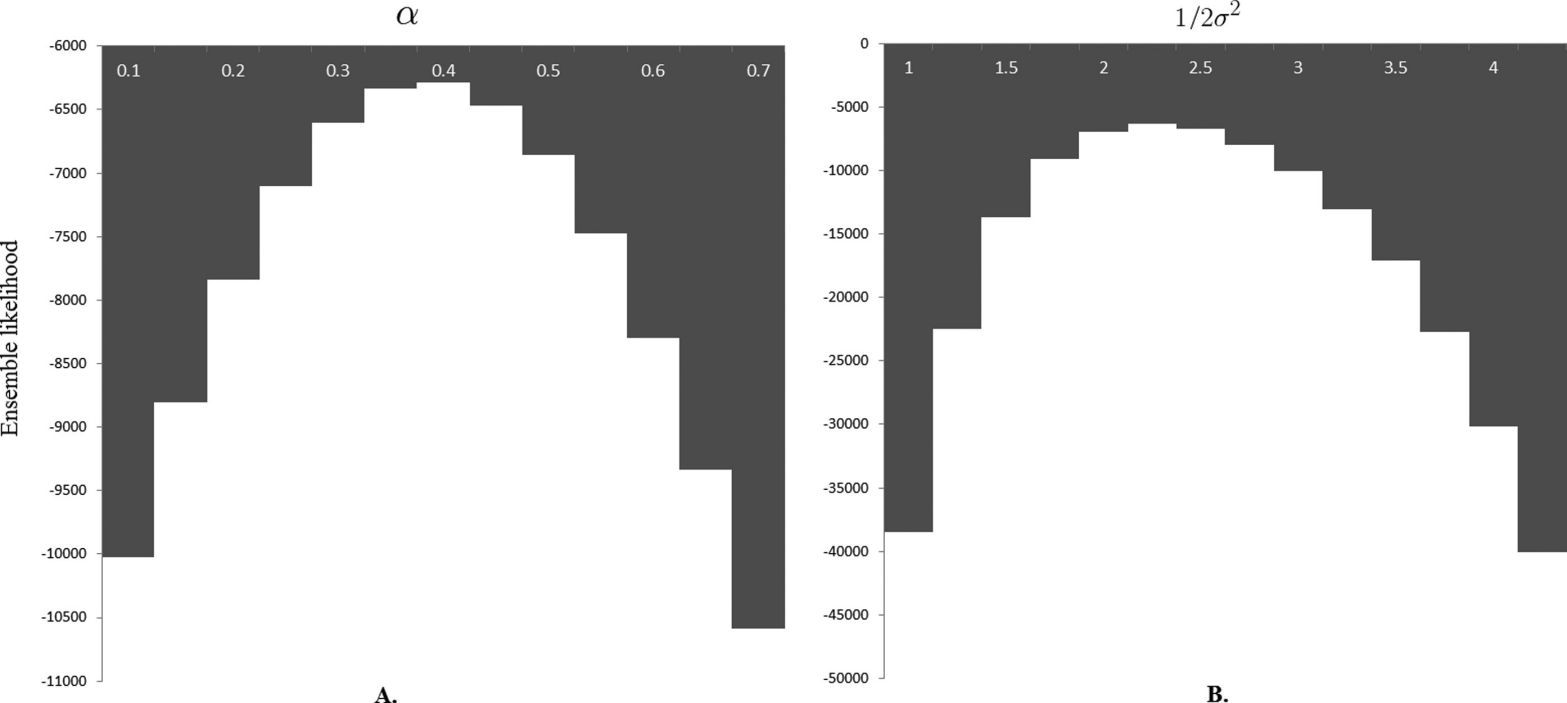
The estimations of parameters α and σ in our Bayesian inferential framework for chromosome 1 of yeast, corresponding to different choices of the parameters. Histograms stand for different values of the ensemble likelihood computed using Equation (15) with respect to different values of the parameter. (**A**) The plot of the ensemble likelihood versus parameters α. The value of 1/2σ^2^ used here was 2.25. (**B**) The plot of the ensemble likelihood versus parameter 1/2σ^2^. The value of α used here was 0.4.

In our Bayesian inference framework for modeling chromatin structures, the parameters, including experimental noise σ and the exponential component α for converting interaction frequencies to spatial distances, can be estimated in a rational way, as described in Algorithm 1. Here, we used the test on chromosome 1 of yeast as an example to demonstrate how our algorithm chooses the best estimates of these parameters. We plotted a distribution of the ensemble likelihood (which was calculated using Equation (15)) versus different choices of the parameters, as shown in Figure 3. The plotted histograms show how different choices of the parameters can affect the overall ensemble likelihood. For both α and $\frac{1}{2\sigma ^{2}}$ parameters, we observed a clear peak, where the ensemble likelihood was maximized. This observation indicated that the optimal values of the parameters in our Bayesian model can be estimated reasonably.

**Figure 4.**
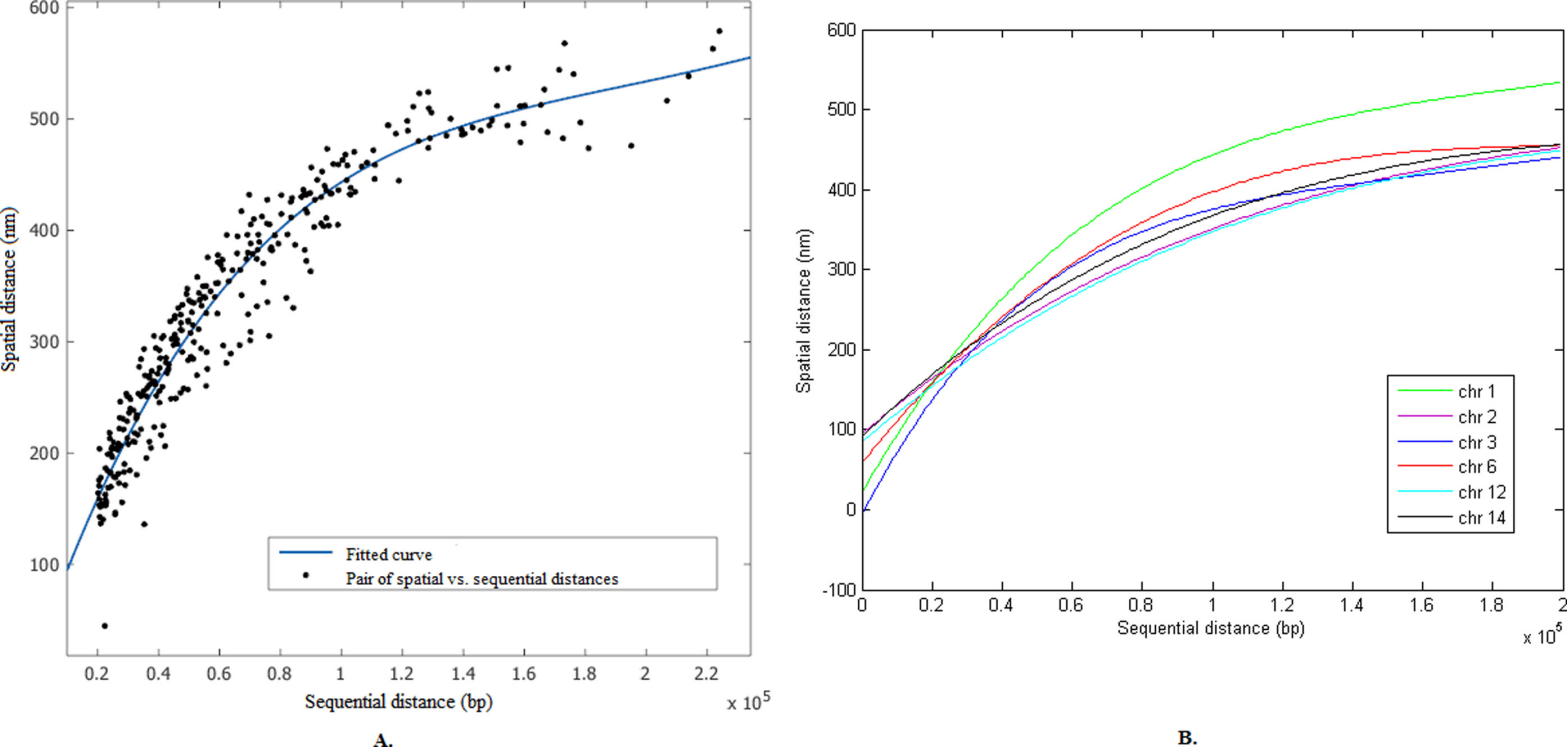
The relationship between spatial and sequential distances. We performed curve fitting for all pairs of genomic loci in the 3C-based data. (**A**) Curve fitting for chromosome 1. (**B**) Fitted curves for different chromosomes to describe how spatial distances change according to sequential distances.

Based on the estimated values of the parameters α and $\frac{1}{2\sigma ^{2}}$, we further investigated the relationship between sequential and spatial distances, which is an important feature for studying the spatial organization of the genome (19,56). In particular, we plotted all pairs of spatial versus sequential distances, and performed curve fitting to find the trend that describes how spatial distances change according to sequential distances. As shown in Figure 4, the spatial distances increase as the corresponding genomic loci are further apart in sequence, but the fitted curves seem to be saturated or increase slowly when the sequential distance is larger than a certain threshold (Figure 4). This observation is consistent with other studies in the literature (19,56). Probably this trend is caused by the size limitation of the nuclei. In Figure 4B, different chromosomes displayed distinct trend curves. This is expected, as the packing of individual chromosomes can be influenced by different settings, such as genomic sizes and interaction densities.

### Validation through cross-validation

**Figure 5.**
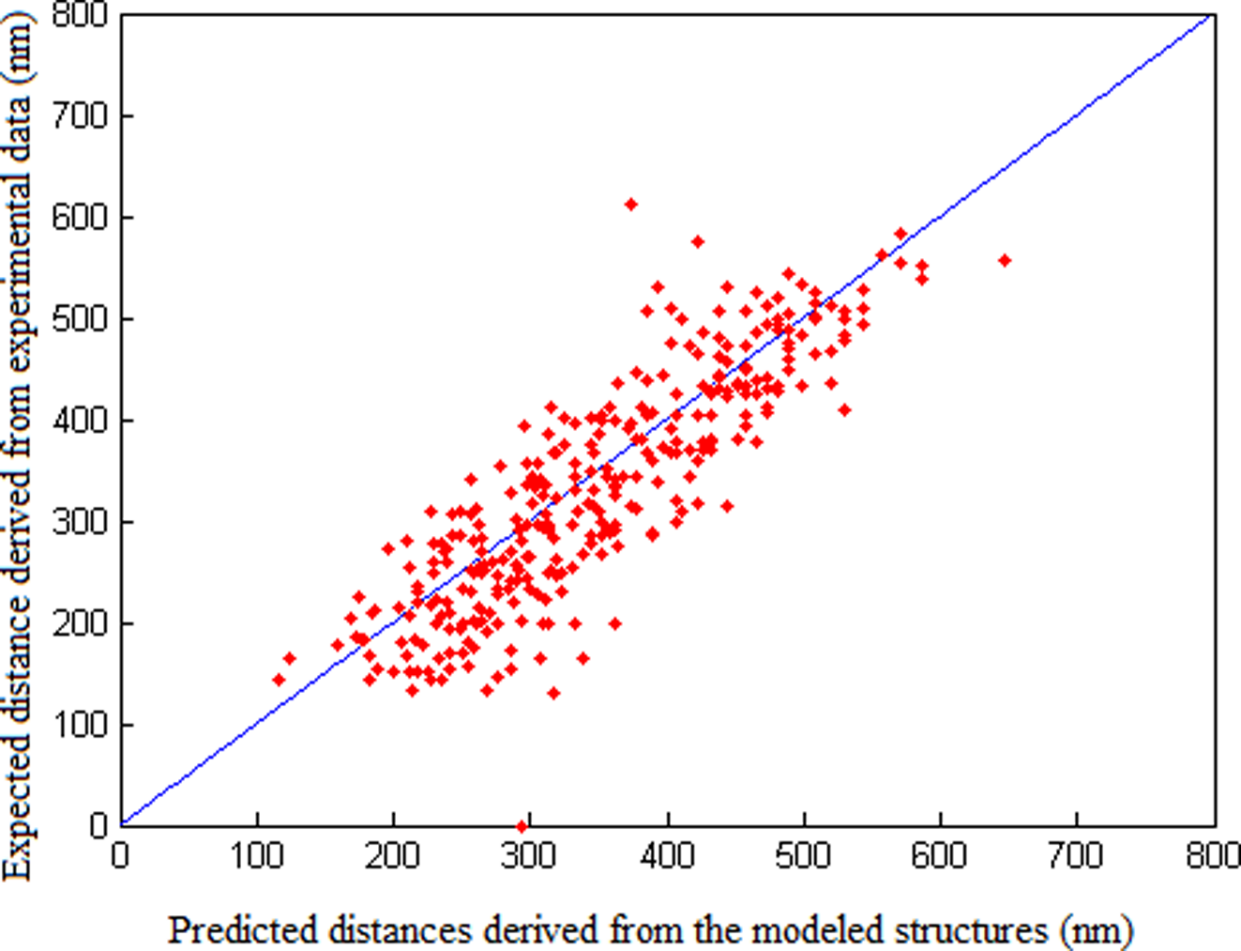
The 10-fold cross-validation results for chromosome 1 of yeast, in which scatter plots of predicted spatial distances derived from the modeled structures versus expected distances converted from interaction frequency data are shown. The correlation is above 0.862. The 5-fold cross-validation shows similar results.

To examine the reliability of the modeled structures and validate the performance of our chromatin structure modeling pipeline, we performed cross-validation to assess the quality of the structure modeling results. We used the test on chromosome 1 of yeast as an example here. The result of 10-fold cross-validation is shown in Figure 5. In the 10-fold cross-validation, we first divided the whole set of the distance constraints converted from the original 3C-based data into 10 subsets, each of approximately equal size. Then we alternatively picked one subset as test data and used the remaining nine subsets as training data. The combined results of the 10-fold process was then used to evaluate the structure modeling results. When evaluating the performance of our algorithm on test data during cross-validation, we used the correlations between the predicted spatial distances derived from the modeled structures (which were computed based on the training data) and the expected distances derived from the data in the test data to measure how well the inferred structures satisfy the experimental data.

As shown in Figure 5, the modeled structures in our ensemble agreed well with the distance constraints converted from 3C-based data, with the scatter plots distributed near the diagonal line and the correlation above 0.86. We performed the same cross-validation procedure for other chromosomes (i.e. chromosomes 3, 6 and 14) of yeast and observed similar results. We also conducted a 5-fold cross-validation which showed similar results. These validation results indicate that our structure modeling approach is able to derive an accurate chromatin structure ensemble that agrees well with the distance restraints converted from 3C-based data.

### Examination of the computed structure ensemble

**Figure 6.**
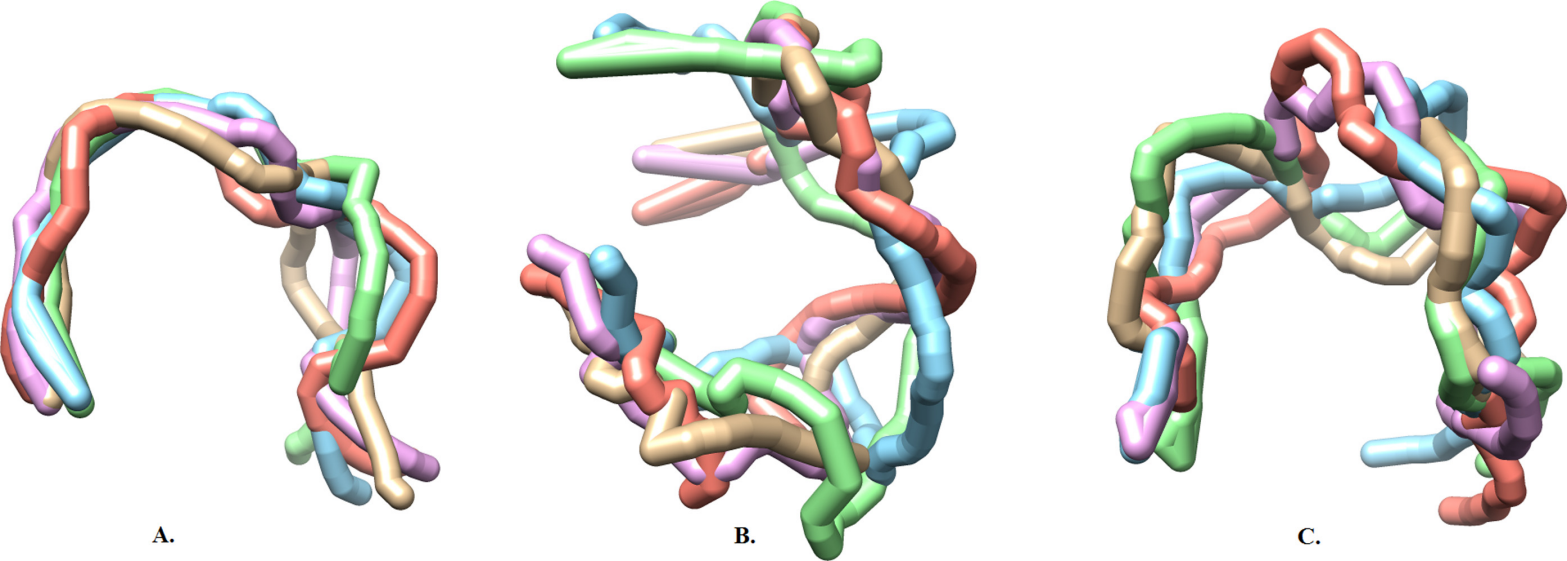
Top five structures with the largest weights in the ensemble inferred from our Bayesian framework. (**A**) Chromosome 1. (**B**) Chromosome 3. (**C**) Chromosome 6. For each chromosome, all structures in the ensemble were aligned using the singular value decomposition (SVD) algorithm (57). The visualization was conducted using UCSF Chimera (58).

In this section, we examined the chromatin structure ensemble modeled from our Bayesian framework. We looked into both individual conformations and their corresponding weights in the ensemble. The quality of the structure ensemble inferred by our Bayesian framework was investigated from different perspectives. We first checked the diversity of the structures in the ensemble. Supplementary Figure S1 gives an example of the weight distribution of individual structures in the ensemble, which shows that the weight of different structures in the ensemble was quite close to each other, but not exactly the same. Figure 6 shows the top five structures with the largest weights in the ensemble for chromosomes 1, 3 and 6, in which all structures had been aligned using the singular value decomposition (SVD) algorithm (57) and the visualization was conducted using UCSF Chimera (58). The structure superimposition showed that individual conformations of the same chromosome were structurally similar. On the other hand, the chromosomes of larger size displayed more structural diversity. For example, chromosomes 3 and 6, which have a larger genome size than chromosome 1, exhibited more structure variation. As shown in Figure 6, a noticeable geometric feature of the 3D structures of chromosomes 3 and 6 is that, two ends of the chromosome interacted with each other in 3D space, thus the whole chromosome formed a loop-like structure, which was consistent with other studies in the literature (59).

We then examined how well the structures in the ensemble fitted the distance restraints converted from experimental data. More specifically, we compared the predicted local distances back-computed from the structures in the ensemble with the spatial distances converted from experimental data. Supplementary Figure S2 shows two examples of the plots of the predicted versus expected spatial distances. In addition, the correlations between predicted and expected spatial distances are shown in Supplementary Figure S3. The comparison results show that the computed structures in the ensemble agreed well with the spatial distances converted from experimental data, with scatter plots distributed near the diagonal line and the Pearson's correlation larger than 0.8.

### Comparisons with other biological observations

In addition to the cross-validation method, we verified the structure modeling results using the known distance constraints derived from other experimental evidence available in the literature. By doing so, we can further rigorously inspect the accuracy of the structure ensemble modeled by our algorithm. We were particularly interested in those pairs of genomic loci which were not observed in the original 3C-based data, and wanted to check whether they can be further validated using other sources of experimental data.

**Figure 7.**
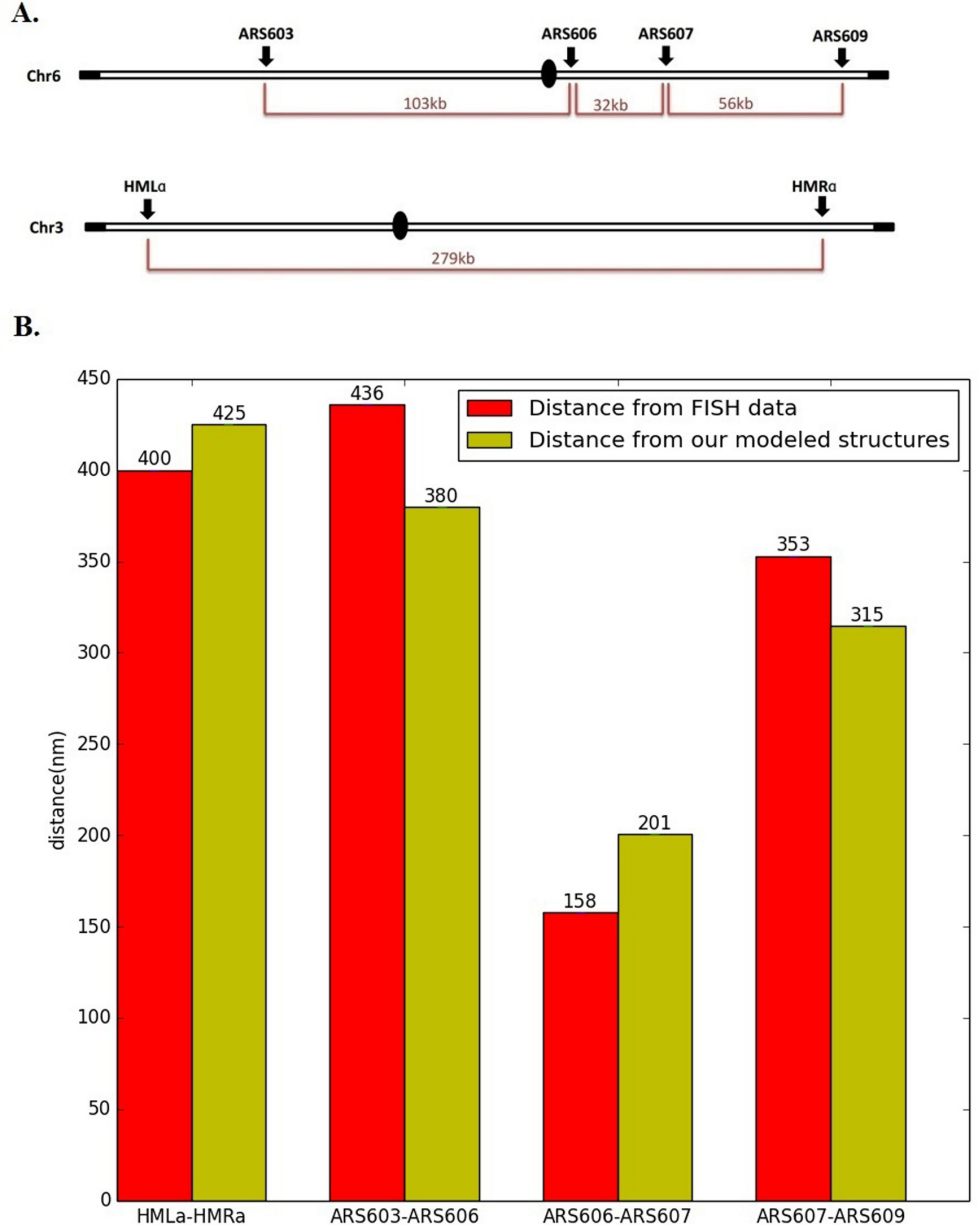
Validation through known spatial distance constraints derived from FISH imaging data. (**A**) Schematic illustration of the locations of the tested genomic loci on chromosomes 3 and 6. (**B**) Comparison between spatial distances derived from our modeled structures and FISH imaging data for four pairs of genomic loci on chromosome 3 and 6. The FISH imaging data were obtained from (56).

We first performed the validation using the FISH imaging data available from the previous studies (56,60). FISH, developed several decades ago, is a traditional method to investigate the spatial arrangements of chromosomes. By using the fluorescent probes bound to certain parts of chromosomes and then imaging them with fluorescence microscopy, the FISH technique is able to detect the relative spatial positions of a specific pair of genomic loci on the chromosomes (61). Despite its low throughput, the sparse set of spatial distance constraints derived from FISH data can still be used to validate our structure modeling results. In particular, we selected four pairs of genomic loci for our validation, in which the spatial distances were derived from the FISH data in (56,60). These pairs of genomic loci included HMLa–HMRa on chromosome 3, and ARS603-ARS606, ARS606-ARS607, ARS607-ARS609 on chromosome 6. Their relative positions along the sequence are shown in Figure 7A. As shown in Figure 7B, the comparison results demonstrate that the corresponding spatial distances in our predicted models agreed well with the FISH observation, with deviation less than 60 nm. Considering that FISH is a fluorescence microscopy-based method with certain experimental noise and uncertainty, the discrepancy with such a small distance range is reasonable and acceptable.

Although HMLa and HMRa are located near two ends of chromosome 3 and far away from each other along the sequence, they were found to have specific long-range interactions (60). Thus, it is expected that they are close to each other in 3D space. The analysis of the 3D FISH imaging data has confirmed this hypothesis and indicated that their spatial distance is less than 500 nm in most occasions (60). Our structure modeling results further confirmed this finding.

We further checked the predicted spatial distances of our modeled chromatin structures using known genetic interactions that have been studied in the literature. The interactions between genes with various functions have been previously observed among the whole genome (62–66). Based on our structure modeling pipeline, we can examine the spatial distances corresponding to these known genetic interactions, and perhaps predict unknown genetic interactions. Here, we selected a list of the most interactive genes (i.e. most frequently interacting with other genes) and checked the spatial distances between corresponding genomic loci in our predicted structures. Noted that although these selected pairs of genomic loci are quite interactive according to the Saccharomyces Genome Database (SGD), their interactions were not directly measured in the original 3C-based data. As listed in Table 2, for the most interactive genes, they had spatial distances within 137–715 nm from their interacting genomic locus partners in our modeled structures. We think this range of spatial distances is reasonably small between the evaluated pairs of genomic loci. Our reasoning is mainly based on the following facts. First, the range of spatial distances 137–715 nm shown in Table 2 is comparable to the relatively reliable measurements from the FISH experiments. Our previous FISH observations show that two genomic loci can have spatial distance near 500 nm, and the best resolution of FISH imaging is 50–100 nm. Thus, a margin of 100–200 nm around 500 nm should be tolerable. Second, the spatial size of the yeast genome is generally beyond 2000 nm (67), which is much larger than the range of the spatial distances between interacting genes listed in Table 2. Third, the gene pairs selected from the SGD functionally interact with each other (68,69). Although a pair of functionally interacting genes is often correlated with physical contact or spatial proximity (70,71), a functional interaction can also be caused by indirect interactions (e.g. regulation through another protein). Thus, it is reasonable to use a slightly larger range of spatial distances indicated from functional interactions between genes as criteria to examine the spatial arrangements of chromosomes.

**Table 2.** Validation of the predicted spatial distances using known genetic interactions in the literature

Gene 1 (ORF)	Chromosome location of gene 1	Gene 2 (ORF)	Chromosome location of gene 2	Predicted spatial distance (nm)	Reference
UBI4	chrXII:64062-65207	HRD3	chrXII:556788-559289	582.8	(63)
UBI4	chrXII:64062-65207	HRT3	chrXII:337265-336231	492.6	(62)
UBI4	chrXII:64062-65207	HSP104	chrXII:88623-91349	137.6	(64)
UBI4	chrXII:64062-65207	HSP60	chrXII:665002-663284	595.0	(62)
UBI4	chrXII:64062-65207	NAM2	chrXII:884751-882067	624.0	(65)
UBI4	chrXII:64062-65207	RPN13	chrXII:965560-965090	714.7	(62)
UBI4	chrXII:64062-65207	SKI2	chrXII:919019-915156	660.0	(65)
UBI4	chrXII:64062-65207	SMD2	chrXII:694378-694800	579.1	(65)
UBI4	chrXII:64062-65207	STP3	chrXII:871697-872728	614.4	(65)
UBI4	chrXII:64062-65207	TOS4	chrXII:522012-520543	559.1	(62)
GIS2	chrXIV:167790-167329	BRE5	chrXIV:718327-716780	565.1	(62)
GIS2	chrXIV:167790-167329	CNM67	chrXIV:224469-222724	309.5	(65)
GIS2	chrXIV:167790-167329	PET8	chrXIV:625829-624975	488.0	(62)
GIS2	chrXIV:167790-167329	POP1	chrXIV:233695-231068	315.8	(65)
GIS2	chrXIV:167790-167329	RIO2	chrXIV:255353-256630	374.0	(65)
GIS2	chrXIV:167790-167329	WHI3	chrXIV:269593-267608	395.0	(65)
HEK2	chrII:160184-161329	PDR3	chrII:217470-220400	274.0	(66)

Information of the interacting genes was acquired from the Saccharomyces Genome Database (SGD). We selected a list of genes (labeled with ‘Gene 1’ in the first column) which interact most frequently with others (labeled with ‘Gene 2’ in the third column) according to the records in the SGD database (3363 interactions recorded in the SGD for UBI4, 1215 for GIS2 and 1130 for HEK2). In addition, the interactions between the selected genomic loci were not directly observed in the original 3C-based data. ORF stands for open reading frame.

Overall, the result in Table 2 indicates that our computed chromatin structures displayed good agreement with the previous studies on genetic interactions. It is true that most of the observed spatial distances indicated in Table 2 are much larger than those in the bead-chain model studies in (34), which is equivalent to 1.5× the Lennard–Jones size parameter. This may be because those pairs of interacting genes with small distances (i.e. near 45 nm) are not detected by the list of functionally interacting ones, or those functionally interacting genes (listed in Table 2) have larger spatial distances. If it is the latter case, one may question whether our original choice (i.e. 30 nm) of the Lennard–Jones size parameter is suitable. However, given our current knowledge about the energy model of a polymer chain model, we still think that 30 nm is an appropriate choice. In our model, although it may be true that the contact distances in the computed structures are much larger than our choice of the Lennard–Jones size parameter, this parameter can be still useful for excluding a large fraction of conformations with spatial collision during the structural modeling process.

We also checked the number of pairs of genomic loci whose expected spatial distance (i.e. the weighted value over all conformations in the ensemble) is less than 100 nm, which is comparable to the contact distance 45 nm suggested in (34). We only focused on those pairs of genomic loci which are at least five segments away from each other along the sequence. Indeed, we found a number of pairs of genomic loci with close distances in our final modeled structures of the yeast chromosomes. For example, we observed 111 and 57 pairs of non-adjacent genomic loci with spatial distances 45–100 nm for chromosomes 2 and 14, respectively. This indicates that the choice of the Lennard–Jones parameter has probably attributed to the final structure modeling results, whereas most of these pairs were not detected by those genetic interactions listed in Table 2.

## CONCLUSION

In this study, we have developed a novel method for chromatin spatial structure modeling based on 3C-based data. We took advantage of the Bayesian inference framework, which allowed us to integrate different types of information with experimental data to determine 3D architectures of chromosomes. With more information, we can achieve more accurate and reliable modeling of chromatin structures. The conformational energy of a polymer chain derived from a polymer model was used as prior information in our Bayesian inference framework. We also proposed an EM-based algorithm to estimate unknown parameters of Bayesian inference model and derive 3D coordinates of chromatin structure. In addition, considering the dynamical feature of chromatin shape, we used a structure ensemble to describe possible states of chromatin organization. We have tested our structure modeling pipeline on real 3C-based data of yeast genome and examined the performance of our structure inference framework. The modeling results were validated through cross-validation and comparisons with previous fluorescent imaging studies. Furthermore, we verified the modeled chromatin structures using known genetic interactions derived from the SGD. The test and validation results indicated that our approach can provide an accurate and promising tool for modeling 3D architectures of chromosomes, which will be useful for further revealing unknown genomic features and understanding complex biological processes of the genome.

## SUPPLEMENTARY DATA

Supplementary Data are available at NAR Online.

SUPPLEMENTARY DATA
