# Supplementary material for: Inferential modeling of 3D chromatin structure
Source: Nucleic Acids Res. 2015 Feb 17;43(8):e54. doi: 10.1093/nar/gkv100 (PMC4417147; doi:10.1093/nar/gkv100)
Supplement: SUPPLEMENTARY DATA [file supp_43_8_e54__index.html]

Inferential modeling of 3D chromatin structure — SUPPLEMENTARY DATA 

# Inferential modeling of 3D chromatin structure

## SUPPLEMENTARY DATA

**Files in this Data Supplement:**

- Supplementary Material
