# Supplementary material for: Inferential modeling of 3D chromatin structure
Source: Nucleic Acids Res. 2015 Feb 17;43(8):e54. doi: 10.1093/nar/gkv100 (PMC4417147; doi:10.1093/nar/gkv100)
Supplement: SUPPLEMENTARY DATA [file supp_gkv100_nar-02979-met-n-2014-File013.pdf]

Supplementary Material for

"Inferential modeling of 3D chromatin structure"

Siyu Wang[1]
Jinbo Xu[2]
Jianyang Zeng[3,4,*]

December 24, 2014

The following is an appendix providing supplementary information to support some claims of the paper. **Supplementary material S1** describes the details of converting original interaction frequency data to spatial distances and the estimation of chromatin size as an restraint. **Supplementary material S2** provides some additional figures that are necessary to substantiate some claims in the paper.

S1 Converting interaction frequencies to spatial distances

One necessary step in our structure modeling pipeline is to transform interaction frequencies to spatial distance restraints between genomic loci. It is a commonly-accepted assumption that the spatial distance between a pair of genomic loci is inversely proportional to the corresponding interaction frequency. In this paper, we follow this assumption that has been widely accepted in the literature of chromatin structure modeling [6, 4]. In particular, we use the following mapping function:

$$D_i = \beta f_i^{-\alpha}, \tag{S1}$$

where f_i and D_i represent the interaction frequency and the converted spatial distance of the i-th data restraint, and α and β represent two parameters that define this mapping function.

In the Methods section of the paper, we have discussed how to compute the exponential factor α. Here, we mainly focus on the determination of coefficient β, assuming that the value of parameter α has been determined. We choose the value of parameter β based on empirical rules about the geometric restraints of a chromosome.

We use d_{mean} to represent average physical distance between genomic loci that can be observed by Hi-C experiments. In principle, the value of d_{mean} should represent the mean value of all spatial distances

[1]Department of Automation, Tsinghua University, Beijing, 100084, P.R. China
[2]Toyota Technological Institute at Chicago, 6045 S Kenwood, IL 60637, USA
[3]Institute for Interdisciplinary Information Sciences, Tsinghua University, Beijing, 100084, P.R. China
[4]MOE Key Laboratory of Bioinformatics, Tsinghua University, Beijing, 100084, P.R. China
[*]Corresponding author: Jianyang Zeng, zengjy321@tsinghua.edu.cn, Tel: +86 010 62781693; Fax: +86 010 62797331.

converted from the detected interaction frequencies recorded in the Hi-C dataset, and its value should be the same for each chromosome. Following the previous literature, we assume that the average spatial distance between a pair of interacting genomic loci is 350nm for yeast, that is, $d_{mean} = 350nm$ [5, 1, 2]. Based on this information, we can easily derive the value of parameter β once the exponential factor α has been determined, that is, $\beta = \frac{h \cdot d_{mean}}{\sum f_i^{-\alpha}}$, where f_i stands for the interaction frequency and h is the total number of data records in the Hi-C data.

We assume that a chromosome is spatially distributed into a cube of size $\varphi \times \varphi \times \varphi$. Here φ is also called the *size of the chromosome*. In general, the size of a certain chromosome φ with sequential length l_s can be expressed as

$$\varphi = \sqrt[3]{\frac{l_s}{l_g}\left(\frac{4}{3}\pi(\frac{1}{2}d)^3\right)}, \tag{S2}$$

where l_g is the whole genome size and d is the diameter of the nucleus. Here, we use the yeast case as an example to demonstrate how to determine the size of a chromosome φ. Suppose that the diameter of the yeast nucleus d is about $2\mu m$. Then the volume of the nucleus is $V = \frac{4}{3}\pi(\frac{1}{2}d)^3 \approx 4.189\mu m^3$. Thus, the volume of chromosome 1 should be $V_1 = \frac{227K}{12.1M}V = 7.86 \times 10^{-2}\mu m^3$ and its size should be $\varphi = \sqrt[3]{V_1} \approx 428nm$. Once φ is determined, we can use this information to help generate the initial structure of a chromosome. Our assumption that a chromosome is arranged in a cube may be less accurate compared to the spindle model in [3]. On the other hand, the cube model is only used as a starting point for modeling chromatin structures. Indeed, to test the robustness of our structure modeling pipeline, we also tried different parameter settings of the initial approximation model, and found that the starting states did not affect much on the final structure modeling results.

S2 Supplementary figures

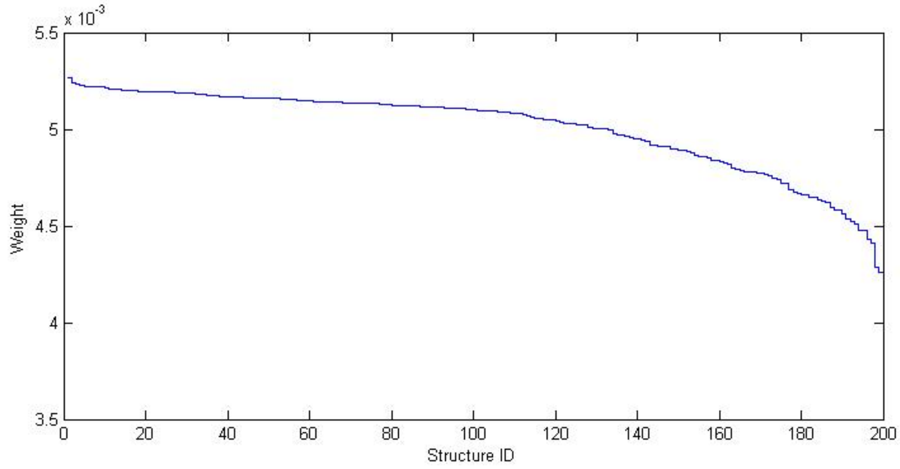

Figure S1: The weight distribution of individual structures in the ensemble for chromosome 1 (the size of ensemble k=200). The structures in the ensemble were sorted according to their weights.

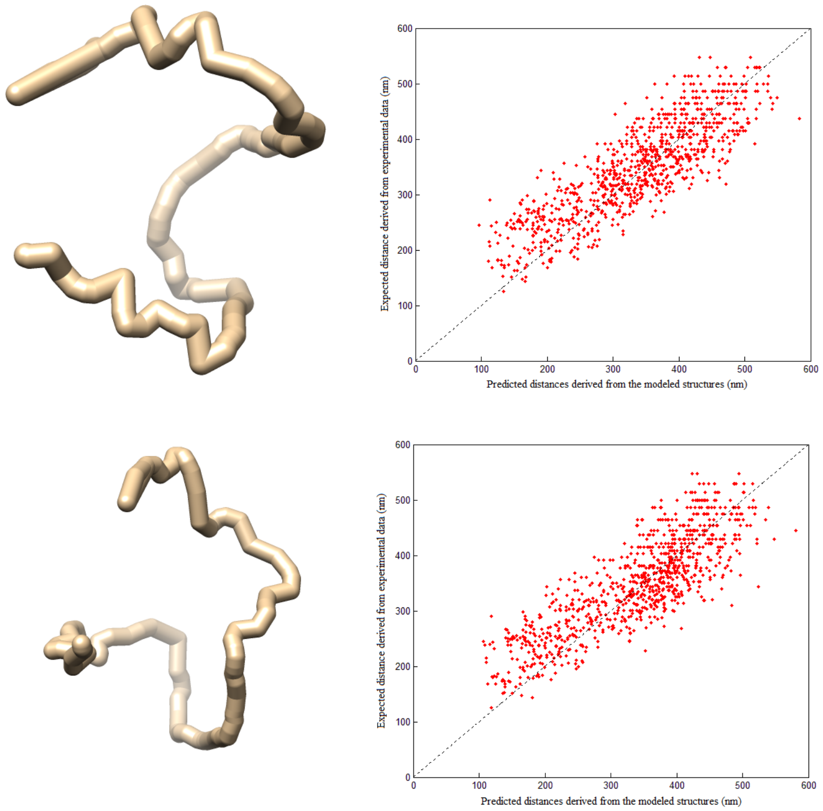

Figure S2: The examination of two individual structures in the ensemble of chromosome 3. The 3D structures are shown on the left, while the plots of the predicted distances derived from the modeled structures vs. expected distances derived from experimental Hi-C data are shown on the right. The correlations between predicted vs. converted spatial distances were 0.847 and 0.837 for the top and bottom structures, respectively.

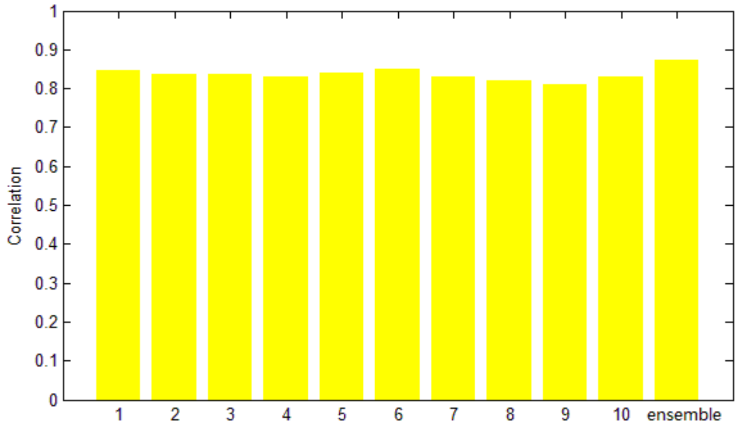

Figure S3: The correlations between the predicted distances derived from the modeled structures and the expected distances between genomic loci derived from experimental observations (i.e., Hi-C data). The first ten bars show the results of the first ten structures with the largest weights, while the last one shows the correlation for all structures in the ensemble. When computing the correlation for the whole ensemble, the predicted distances between genomic loci were calculated based on the ensemble-averaged values.

Suplementary References

[1] Kerstin Bystricky, Patrick Heun, Lutz Gehlen, Jörg Langowski, and Susan M Gasser. Long-range compaction and flexibility of interphase chromatin in budding yeast analyzed by high-resolution imaging techniques. *Proceedings of the National Academy of Sciences of the United States of America*, 101(47):16495–16500, 2004.

[2] Job Dekker. Mapping in vivo chromatin interactions in yeast suggests an extended chromatin fiber with regional variation in compaction. *Journal of Biological Chemistry*, 283(50):34532–34540, 2008.

[3] Zhijun Duan, Mirela Andronescu, Kevin Schutz, Sean McIlwain, Yoo Jung Kim, Choli Lee, Jay Shendure, Stanley Fields, C Anthony Blau, and William S Noble. A three-dimensional model of the yeast genome. *Nature*, 465(7296):363–367, 2010.

[4] James Fraser, Mathieu Rousseau, Solomon Shenker, Maria A Ferraiuolo, Yoshihide Hayashizaki, Mathieu Blanchette, and Josée Dostie. Chromatin conformation signatures of cellular differentiation. *Genome Biol*, 10(4):R37, 2009.

[5] Adriana Miele, Kerstin Bystricky, and Job Dekker. Yeast silent mating type loci form heterochromatic clusters through silencer protein-dependent long-range interactions. *PLoS genetics*, 5(5):e1000478, 2009.

[6] Mathieu Rousseau, James Fraser, Maria A Ferraiuolo, Josée Dostie, and Mathieu Blanchette. Three-dimensional modeling of chromatin structure from interaction frequency data using Markov chain Monte Carlo sampling. *BMC bioinformatics*, 12(1):414, 2011.
